# Complete Genome Sequences of 24 Strains of Bacillus cereus Isolated from Nosocomial Infection and Bacteremia Cases in Japan

**DOI:** 10.1128/mra.01203-21

**Published:** 2022-03-15

**Authors:** Yoshikazu Furuta, Mai Tsujinouchi, Misheck Shawa, Tuvshinzaya Zorigt, Yuya Miyajima, Atmika Paudel, Satowa Suzuki, Hideaki Higashi

**Affiliations:** a Division of Infection and Immunity, International Institute for Zoonosis Control, Hokkaido University, Sapporo, Hokkaido, Japan; b Antimicrobial Resistance Research Center, National Institute of Infectious Diseases, Tokyo, Japan; University of Rochester School of Medicine and Dentistry

## Abstract

Bacillus cereus is mainly associated with foodborne illness but sometimes causes nosocomial infections. We previously reported that B. cereus strains of a specific sequence type, ST1420, were associated with nosocomial infection. Here, we determined the complete genome sequences of B. cereus strains isolated from nosocomial infection cases in Japanese hospitals.

## ANNOUNCEMENT

Bacillus cereus is a Gram-positive bacterium that typically causes foodborne illness characterized by vomiting and diarrhea (1). Especially in immunocompromised patients, the species also causes severe nongastrointestinal infections such as bacteremia, endocarditis, meningoencephalitis, and pneumonia, resulting in nosocomial infection in some cases (2–8). We previously analyzed the B. cereus strains isolated from nosocomial infection cases in three hospitals in Japan using multilocus sequence typing and revealed that strains of the specific novel sequence type 1420 (ST1420) were present in all three hospitals (9). As the cases occurred in different years and all hospitals were distantly located from each other, the result suggested the ST1420 could be a prevalent ST in recent B. cereus nosocomial infection cases in Japan (9).

From each of the three hospitals, we chose 4 ST1420 and 4 non-ST1420 strains and conducted whole-genome sequencing (9). Each strain was first inoculated into lysogeny broth (LB) agar plates from a glycerol stock and incubated at 37°C for 16 h. Next, a single colony was picked into 2 mL of LB medium and was grown by shaking at 144 rpm at 37°C for 16 h. Genomic DNA was isolated from the liquid culture using the QIAamp PowerFecal Pro DNA kit (Qiagen). Sequencing libraries for short reads were constructed using Nextera XT DNA library prep kit (Illumina) and sequenced using MiSeq reagent kit v3 (600 cycles) and MiSeq (Illumina), producing 300-bp paired-end reads. For long-read sequencing, genomic DNA was not sheared nor size selected and directly used for the construction of libraries using ligation sequencing kit (catalog no. SQK-LSK109, Oxford Nanopore Technologies) with indexing by native barcoding expansion kit (catalog nos. EXP-NBD104 and EXP-NBD114) and sequenced using MinION with R9.4.1 flow cells. *N*_50_ values were distributed from 7 to 13 kb for each strain.

Short reads produced by MiSeq were first filtered with Trim Galore v0.4.2 for trimming adapter sequences and quality filtering with --paired and --nextera options. Long reads were basecalled using Guppy v3.3.0 and used for *de novo* assembly using Canu v1.8 (10) with options of “corMhapOptions=--threshold 0.8 --ordered-sketch-size 1000 --ordered-kmer-size 14” and “correctedErrorRate=0.105 corOutCoverage=1000.” Assembled contigs were polished with trimmed short reads using Pilon v1.23 with the default parameters (11). Repeats at both ends of contigs were detected by dotplot using Gepard (12), and the repeats were removed manually for circularization.

Genome sequences were annotated and rotated using DFAST v1.2.5 with options of “--organism ‘Bacillus cereus’ --complete t --fix_origin -seq_types chromosome,plasmid --seq_topologies circular,circular --step 1” (13). All raw reads and complete genome sequence data were deposited into DDBJ, with accession numbers and other details shown in Table 1. All strains possessed a chromosome of about 6 Mbp and also possessed 1 to 12 plasmids. Genome comparison analysis of these strains with other strains of the Bacillus cereus group is currently underway and will be reported in subsequent reports.

**TABLE 1:** Accession numbers of sequenced Bacillus cereus strains

Strain	Strain name in reference 9^*a*^	Place of isolation	ST^*b*^	Data for:								G+C content (%)
				MinION reads			MiSeq reads		Complete genome			
				SRA accession no.	No. of reads	*N*_50_ (bp)	SRA accession no.	No. of read pairs	GenBank accession nos.	No. of plasmids	Genome size (bp)	
MRY14-0045	TokyoID2	Patient blood	1420	DRR206386	144,004	13,360	DRR206362	1,282,452	AP022857, AP022858	1	5,669,919	35.3
MRY14-0057	TokyoID14	Patient blood	1420	DRR206387	182,669	13,309	DRR206363	1,179,532	AP022874–AP022876	2	5,684,802	35.3
MRY14-0060	TokyoID17	Patient blood	1421	DRR206388	213,391	10,085	DRR206364	1,167,598	AP022877–AP022885	8	5,778,916	35.5
MRY14-0074	TokyoID21	Towel	365	DRR206389	383,096	8,015	DRR206365	1,097,802	AP022886–AP022893	7	5,473,594	35.4
MRY14-0075	TokyoID22	Towel	26	DRR206390	331,541	9,233	DRR206366	1,118,288	AP022894–AP022902	8	6,021,332	35.4
MRY14-0079	TokyoID23	Towel	1420	DRR206391	229,528	11,841	DRR206367	813,632	AP022903–AP022906	3	5,689,809	35.3
MRY14-0100	TokyoID27	Towel	1429	DRR206392	368,960	8,220	DRR206368	1,087,651	AP022907–AP022914	7	5,651,802	35.5
MRY14-0105	TokyoID29	Microwave oven	1420	DRR206393	222,812	10,404	DRR206369	1,469,094	AP022915–AP022920	5	5,700,385	35.3
J1	TochigiID1	Infusion	1420	DRR206394	182,392	11,671	DRR206370	1,207,265	AP022921–AP022926	5	5,736,497	35.3
J2	TochigiID2	Infusion	365	DRR206395	210,109	11,509	DRR206371	935,634	AP022927–AP022933	6	5,887,179	35.5
J7	TochigiID7	Patient skin	167	DRR206396	302,930	11,457	DRR206372	1,019,824	AP022934–AP022945	11	5,886,752	35.4
J10	TochigiID10	Sheet	999	DRR206397	163,871	12,243	DRR206373	1,016,148	AP022946–AP022951	5	5,713,479	35.3
J39	TochigiID39	Patient blood	1420	DRR206398	370,176	10,522	DRR206374	1,158,552	AP022952–AP022955	3	5,682,193	35.4
J51	TochigiID51	Patient blood	1420	DRR206399	280,606	11,071	DRR206375	988,300	AP022956–AP022963	7	5,788,209	35.3
J62	TochigiID62	Linen room washed towel	167	DRR206400	547,425	7,672	DRR206376	1,015,084	AP022964–AP022969	5	5,808,641	35.4
J75	TochigiID75	Linen room washed towel	1420	DRR206401	273,259	10,947	DRR206377	1,225,232	AP022970–AP022974	4	5,675,732	35.4
30040	Tottori30040	Patient blood	1420	DRR206402	266,594	10,658	DRR206378	1,097,590	AP022975–AP022977	2	5,373,819	35.3
30043	Tottori30043	Patient blood	1420	DRR206403	266,185	10,390	DRR206379	867,430	AP022978–AP022980	2	5,373,258	35.4
30048	Tottori30048	Patient blood	1431	DRR206404	591,277	7,221	DRR206380	825,008	AP022981–AP022985	4	5,786,323	35.3
30052	Tottori30052	Patient blood	163	DRR206405	458,967	7,594	DRR206381	900,636	AP022986–AP022993	7	5,961,792	35.4
30075	Tottori30075	Patient blood	1420	DRR206406	381,379	9,606	DRR206382	1,088,785	AP022994–AP022999	5	5,874,761	35.2
30077	Tottori30077	Patient blood	1420	DRR206407	276,354	12,769	DRR206383	928,363	AP023000–AP023004	4	5,674,353	35.3
30090	Tottori30090	Patient blood	368	DRR206408	605,803	8,348	DRR206384	1,022,484	AP023005–AP023013	8	5,751,514	35.6
30102	Tottori30102	Patient blood	953	DRR206409	490,333	9,087	DRR206385	1,309,287	AP023014–AP023026	12	6,018,453	35.5

a Names of strains used in reference 9. Names start with the name of the place of the hospital where the strain was isolated.

b Sequence type according to the multilocus sequence typing conducted in reference 9.

### Data availability.

Genome accession numbers in public databases are listed in Table 1.
